# Air-Spun Silk-Based Micro-/Nanofibers and Thin Films for Drug Delivery

**DOI:** 10.3390/ijms22179588

**Published:** 2021-09-03

**Authors:** Christopher R. Gough, Xiao Hu

**Affiliations:** 1 Department of Physics and Astronomy, Rowan University, Glassboro, NJ 08028, USA; goughc2@students.rowan.edu; 2 Department of Chemistry and Biochemistry, Rowan University, Glassboro, NJ 08028, USA; 3 Department of Biomedical Engineering, Rowan University, Glassboro, NJ 08028, USA; 4 Department of Molecular and Cellular Biosciences, Rowan University, Glassboro, NJ 08028, USA

**Keywords:** silk fibroin, air spray, film, fiber, crystalline structure, drug release

## Abstract

Micro-/nanofibers have shown high promise as drug delivery vehicles due to their high porosity and surface-area-to-volume ratio. The current study utilizes air-spraying, a novel fiber fabrication technique, to create silk micro-/nanofibers without the need for a high voltage power source. Air-spraying was used to create silk fibrous mats embedded with several model drugs with high efficiency. In order to compare the effect of biomaterial geometry on the release of the model drugs, silk films were also created and characterized. Fourier-transform infrared spectroscopy (FTIR), scanning electron microscope (SEM), differential scanning calorimetry (DSC), thermogravimetric analysis (TGA), and a drug release study were performed on both fiber and film samples to study how the model drugs interact with the protein structure. FTIR analysis showed that while drugs could interact with the protein structure of porous silk fibers, they could not interact with the flat geometry of silk films. As a result, fibers could protect select model drugs from thermal degradation and slow their release from the fiber network with more control than the silk films. A trend was also revealed where hydrophobic drugs were better protected and had a slower release than hydrophilic drugs. The results suggest that the physical and chemical properties of drugs and protein-based biomaterials are important for creating drug delivery vehicles with tailored release profiles and that fibers provide better tunability than films do.

## 1. Introduction

Protein-based drug delivery vehicles are an emerging field aiming to fix many of the problems with current drug delivery methods [1]. Often, these vehicles take the form of micro-/nanofibers [1,2,3,4,5,6,7,8] or thin films [1,2,3,5,6,9]. These types of biomaterials provide several benefits over traditional systemic delivery methods by reducing off-target effects and improving patient compliance with lower dosage requirements [1,10]. Micro-/nanofibers are of particular interest in this study as their high porosity and surface-area-to-volume ratio allows for the efficient loading and release of various therapeutic molecules [6,8,11,12,13]. Protein-based fibers provide the additional benefit of functionalization based on protein structure and amino acid composition in order to improve their biochemical and biophysical interactions with other molecules [14,15]. Currently, the clinical use of submicrometer fibers is limited by a lack of quantitative data and a reliable large-scale production method [16].

A plausible answer for the reliable scale-up of micro-/nanofiber production is through air-spraying. Traditionally, most submicrometer fiber fabrication utilizes electrospinning or techniques related to it [17,18,19,20,21]. Through this method, a polymer solution is spun into fibers by connecting the outlet of a spinneret to a high voltage power source. As electrostatic forces overpower surface tension in the polymer solution, the polymer is drawn into fibers that are collected onto a grounded surface or a collection bath (wet spinning) [22]. The entire process is slow with a low yield, and the need for a high voltage power source makes it a costly and dangerous fabrication method. An alternative utilized in this study is air-spraying or solution spraying. In this method, compressed air is used as the driving force of fiber formation instead of electrostatics [6,11,23,24]. The polymer solution is injected into a concentric nozzle which is fed high pressure compressed air from an air compressor. This high-pressure air provides a shearing force to the polymer solution in order to create strands of fibers; once these strands leave the concentric nozzle, the solvent quickly evaporates, leaving behind a fibrous mat on a collection plate. Air-spraying has shown to have higher throughput than electrospinning [23] making it a promising method for scale-ups as micro-/nanofiber research becomes more clinically relevant.

While fibers have been created from a wide variety of proteins and biomacromolecules, Bombyx *mori* silk was chosen for this study. Silk has a long history in fabrics and biomaterials due to its strong mechanical and thermal properties, chemical stability, and good biocompatibility [25]. Natural silk consists of silk fibroin protein and sticky sericin proteins; with proper preparation, silk fibroin can be separated from the sericin and be used to produce biomaterials in several different forms [20,26,27,28,29,30,31,32], including biomimetic fibers by solution blow spinning [27]. Bombyx *mori* silk in particular contains the amino acid sequence GAGAGS, resulting in the self-assembly of intermolecular beta sheets that give silk its strong mechanical properties [33]. The beta sheet structure of silk also provides bioactivity, making it possible to engineer silk biomaterials with tunable properties and specific behaviors, including controlled drug delivery [34,35].

While the above studies focus on using silk films for controlled drug delivery, there has been little research on the use of silk micro-/nanofibers for the same purpose thus far. Given the general potential of fibers in drug delivery and the ability of silk crystallinity (beta sheets) to naturally govern the release of model drugs, this study sought to compare the biomaterial geometry of 1D silk micro-/nanofibers to 2D silk films. A diverse set of model drugs with varying weight, charge, hydrophobicity, and solubility was chosen to represent the wide variety of drugs in the pharmacopeia today. The effect of model drugs on the protein structure of silk biomaterials and their subsequent release from the materials was studied through FTIR, SEM, TGA, and DSC characterization as well as a drug release study.

## 2. Results and Discussion

### 2.1. Structural Characterization

The structure of silk fibers with and without embedded model drugs was first studied using FTIR. All spectra look relatively the same at the higher wavenumber region. And all samples showed a lack of a formic acid peak, indicating that all excess formic acid was removed during fabrication or drying.

**Figure 1:**
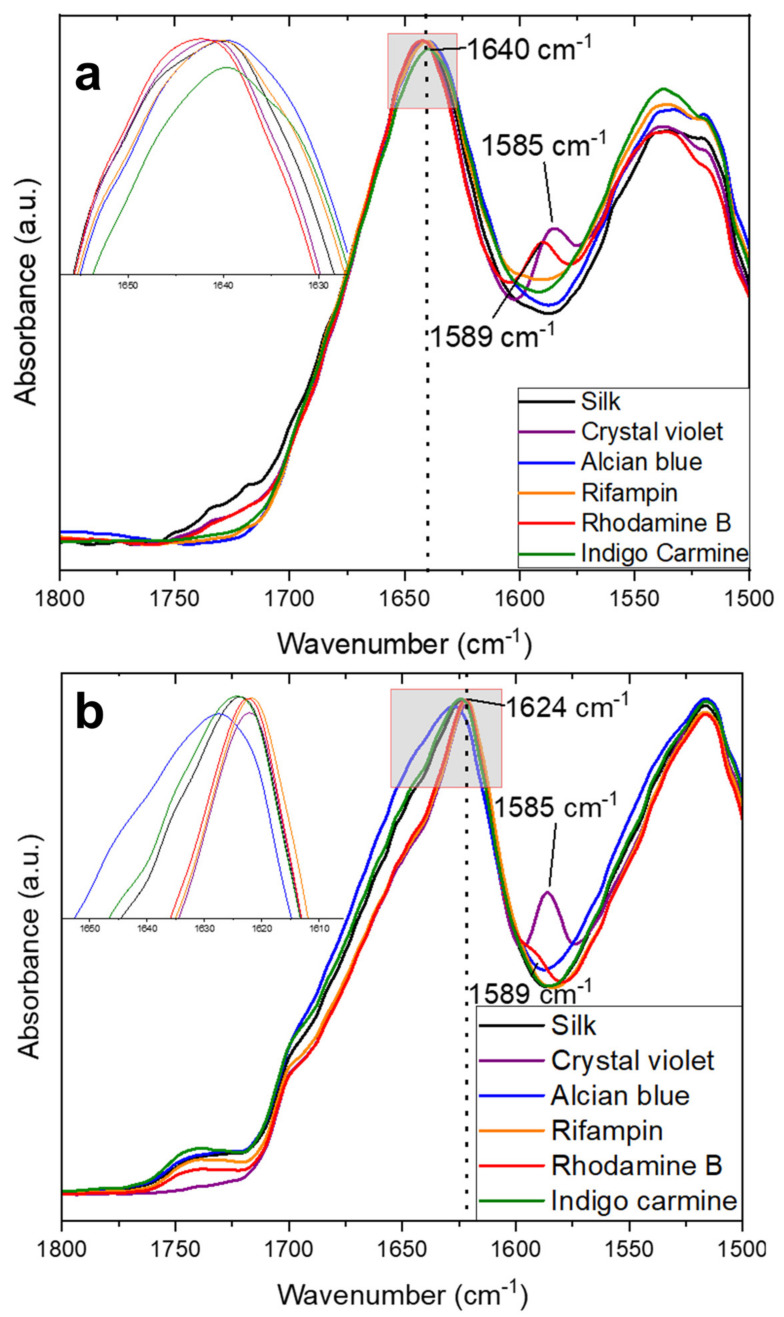
FTIR spectra of the Amide I and II regions of air-spun silk fibers (**a**) before and (**b**) after release of model drugs.

To better understand how the fabrication of the materials and the addition of model drugs impact the protein structure, the Amide I and II regions are highlighted specifically in Figure 1a. This region is commonly used to analyze protein secondary structures [36]. The amide I region, from 1700–1600 cm^−1^, comes from mainly C=O stretching vibrations in the protein backbone, with some minor contribution from N-H in-plane stretching, out-of-phase C≡N stretching, and C-C≡N deformation. The Amide II region is assigned to the 1600–1500 cm^−1^ region, where absorption is caused by an out-of-phase combination of vibrations of C–N stretching and N–H in-plane bending [31]. In these regions pure silk fibers center their absorbance peak at 1639 cm^−1^, indicative of intramolecular β-sheets suspended in a random coil network [37,38,39,40,41,42]. Some model drugs are able to shift the structure slightly towards more β-sheet content: alcian blue and rhodamine B shift the absorbance peak to 1641 cm^−1^ and rifampin shifts this peak to 1640 cm^−1^. Crystal violet and rhodamine B also introduce small shoulders at 1585 cm^−1^ and 1589 cm^−1^, respectively. These shoulders come from the model drugs themselves as they are seen on FTIR spectra of solutions of the molecules [6]. Some model drugs also introduce a shoulder at 1720 cm^−1^ which is likely due to C=O stretching between the protein backbone and the model drug molecules [43,44]. Crystal violet and rhodamine B in particular have high log P values of 1.4 and 1.95 respectively, while maintaining a small size, unlike alcian blue. Because of this hydrophobic nature, these molecules can influence the hydrophobic effect that stabilizes the secondary protein structure of silk. Amine additives have been shown to have complex interactions in the suppression or assistance of protein interactions [45] that likely cause the interactions with the silk side chain seen in this study. Alcian blue is unable to interact in the same way because it is such a large molecule that it is too big to interact with the submicrometer fiber protein structure [6]. Understanding how the physicochemical nature of various therapeutics can affect the protein folding of biomaterials can be a useful tool for creating fine-tuned drug delivery vehicles for a wide variety of purposes.

Upon release of the model drugs after 4 days of soaking in water, the FTIR spectra of the samples were re-analyzed. It is important to preface this analysis with the fact that soaking in water also causes the release of CaCl_2_ ions introduced by the solvent, which causes silk to re-form its native intermolecular beta sheet structure [22,31,46]. This was also the case in this study as shown in the FTIR spectra (Figure 1b). After soaking, all samples convert to an intermolecular beta sheet structure based on the absorbance peaks between 1622–1628 cm^−1^ [31,37]. In addition, the characteristic peaks of crystal violet and rhodamine B at 1585 cm^−1^ and 1589 cm^−1^, respectively, remain but at a reduced intensity because the majority of the molecules have been released from the fibers. The slight variance in this absorbance peak is due to interactions between model drug molecules and the silk protein structure. While pure silk has an absorbance peak centered at 1624 cm^−1^, this peak shifts towards lower β-sheet content with alcian blue shifting this peak to 1628 cm^−1^ or towards higher β-sheet content with rifampin, rhodamine B, and indigo carmine shifting this peak to 1622 cm^−1^. This interaction implies that by finding compatible molecules and proteins, biomaterials with stronger mechanical and thermal properties, or with fine-tuned release mechanisms, can be fabricated by understanding these interactions. Coupled with high porosity, malleability, and a high surface-area-to-volume ratio, micro-/nanofibers have high potential in drug delivery applications, through the study of protein-pharmaceutical interactions.

FTIR analyses of the silk and silk-model drug films revealed a similar result. The full spectra are very similar to that of the fiber samples. Specifically, there is a lack of the formic acid absorbance peak, again indicating that all excess solvent was removed during fabrication of drying before the samples were analyzed.

**Figure 2:**
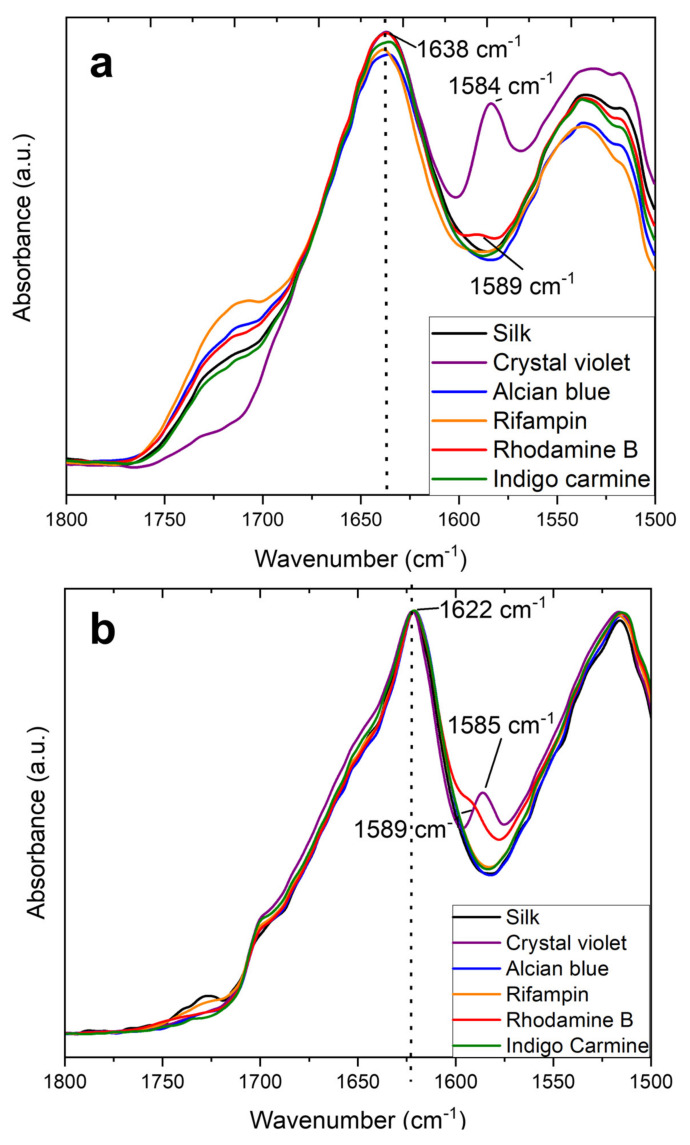
FTIR spectra of the Amide I and II regions of silk thin films (**a**) before and (**b**) after release of model drugs.

A better understanding of the protein structure within the films is highlighted by looking at the Amide I and Amide II regions in Figure 2. The pre-release spectra are shown in Figure 2a and indicate similar trends to the fiber samples with a peak at 1640 cm-^1^ indicative of mainly random coils and some intramolecular β-sheets. Again, crystal violet shows a characteristic peak at 1584 cm^−1^ and rhodamine B shows a characteristic shoulder at 1589 cm^−1^. One area where film samples differ from fibers is that there is no variability in the absorbance peak at 1640 cm^−1^. In fiber samples the model drugs are able to interact with the silk protein structure in the 1D fiber geometry; in the larger, 2D film geometry, these interactions do not happen, so the variations in peak absorbance that were seen in the fibers are not seen in the films. Instead, all samples match the exact absorbance peak of pure silk films. This lack of interaction leads to poorer thermal stability and different release profiles, which will be explained further in their respective sections.

After the model drugs are released through water soaking, all film samples shift towards an intermolecular beta sheet structure indicated by an absorbance peak at 1622 cm^−1^ (Figure 2b). Because the model drugs do not interact with the film protein structure, there is no hindrance in silk self-folding into intermolecular β-sheets once the CaCl_2_ ions are removed. Again, similar to the samples before model drug release, all peaks center at the same wavelength in film samples due to geometric constraints. This differs from the fiber samples where some model drugs could induce higher or lower β-sheet content within the materials.

### 2.2. Thermal Analysis

**Figure 3:**
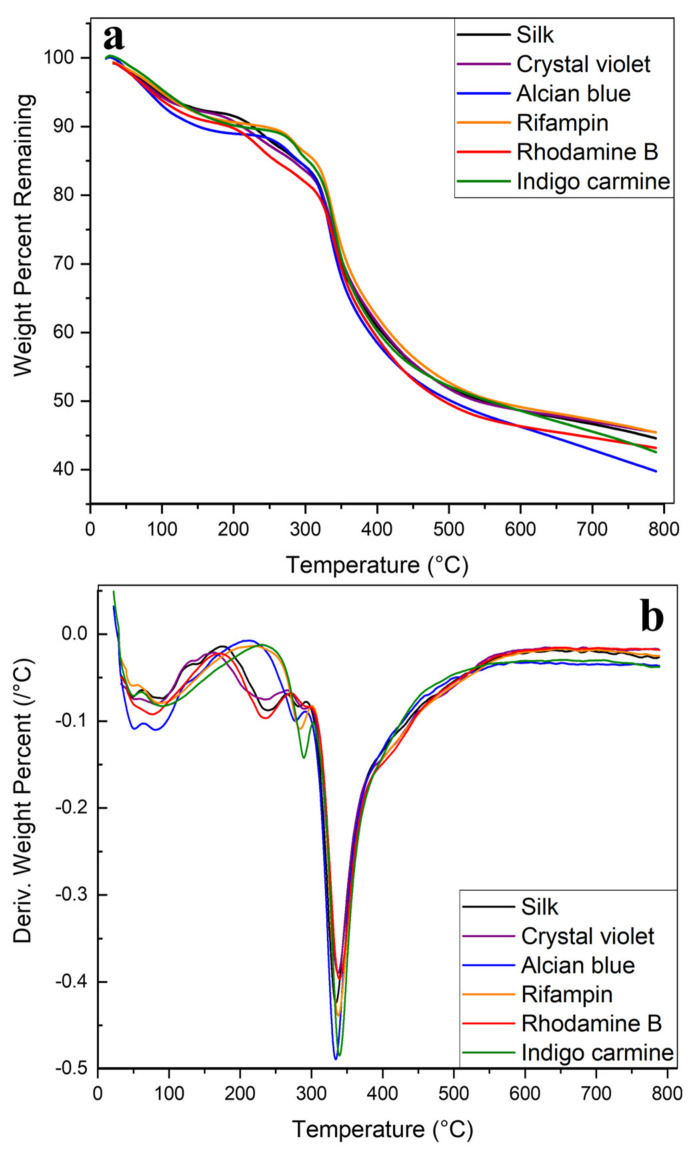
TGA thermograms of silk and silk-model drug air-spun fibers. (**a**) Remaining weight percent of silk and silk-model drug fibers as they were heated to 800 °C. (**b**) Derivative of remaining weight percentage curves.

The overall thermal integrity of silk films and fibers was first studied using TGA. The effects of the model drugs on thermal integrity were also studied. Figure 3 depicts the thermograms produced from analysis on the silk and silk-model drug fibers. In Figure 3a all samples see an initial small mass loss under 100 °C as excess water and solvent embedded in the samples is removed. Fibers then have a gradual degradation between 200–325 °C before major degradation begins. This continues until the 500 °C+ region when degradation slows until 40–50% of the total mass is lost at 800 °C.

Model drugs have varied effects on the thermal stability of the fibers. Rifampin and indigo carmine, which both showed the ability to influence protein structure towards a higher β-sheet content in the FTIR analysis, both push the onset of major degradation in the samples from 230 °C to 280 °C. Rhodamine B, which affected the protein structure the same way as rifampin and indigo blue, does not push the onset of degradation further, but shows a different stabilizing effect by slowing the rate of degradation in the 200–325 °C region. Figure 3b shows the derivative of the TGA curves in Figure 3a. Alcian blue, which also showed an influence on the protein structure in the FTIR analysis, also pushes the onset of degradation and slows the degradation rate compared with silk without any model drugs. After the initial mass loss due to solvent evaporation, alcian blue fibers do not begin degrading until 280 °C compared with 230 °C in silk alone. Crystal violet or rhodamine B does not push initial degradation to a higher temperature but does slow the degradation rate in the major degradation region (350 °C), showing a shallower and broader peak.

**Figure 4:**
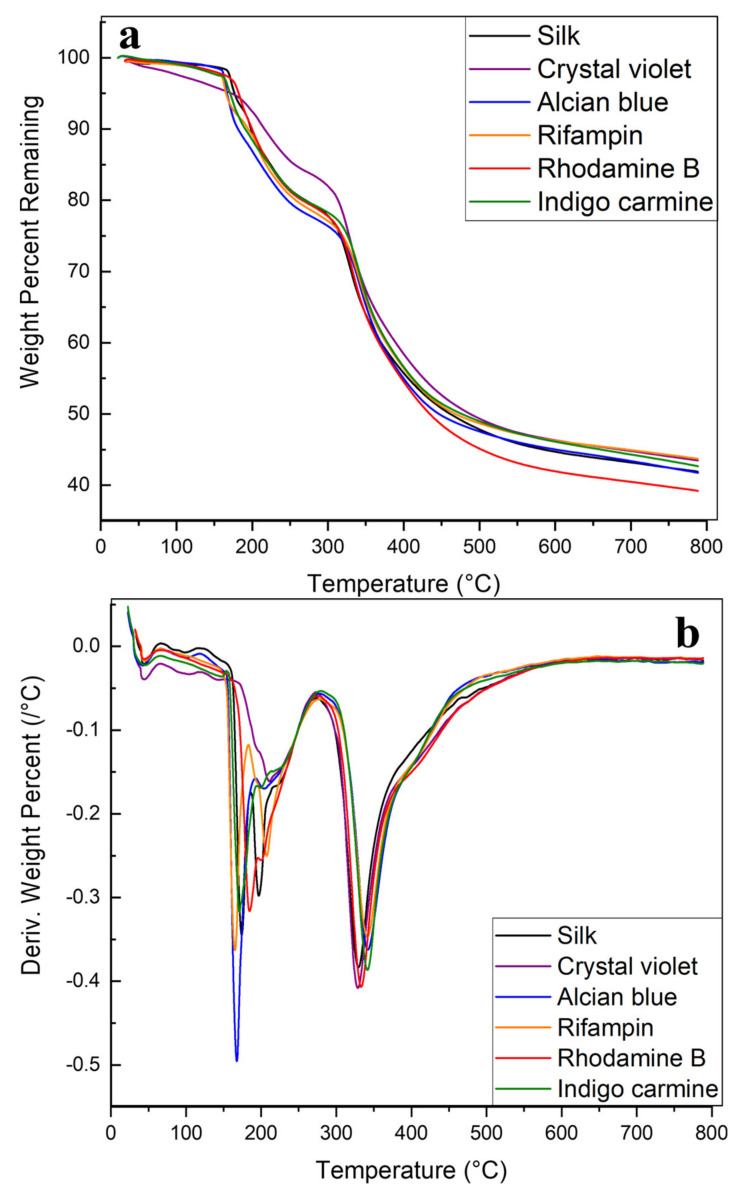
TGA thermograms of silk and silk-model drug thin films. (**a**) Remaining weight percent of silk and silk-model drug thin films as they were heated to 800 °C. (**b**) Derivative of remaining weight percentage curves.

A parallel analysis was also performed on silk films with and without embedded model drugs. Overall, film samples show lower stability than fiber samples. Figure 4 depicts this through the early onset of degradation. There is less degradation under 100 °C in film samples because any excess solvent not evaporated during drying is captured inside of the 2D film geometry, but past this, all samples with or without model drugs begin degrading at 180 °C. This initial mass decrease is also much more rapid in films than in fibers, which is most clearly depicted by the sharp peaks around 180 °C in Figure 4b. Crystal violet films resist this rapid degradation best, instead having slow, constant degradation from 0 to 200 °C when films begin to degrade. This sharp degradation at lower temperatures implies that 2D film geometry is unable to protect the embedded drugs from thermal decomposition in the way 1D fibers could. All film samples still begin degrading earlier than their fiber counterparts, indicating poor thermal integrity. The same is true for major degradation, which begins at 300 °C in films compared with 325 °C in fibers. In the major degradation region (above 325 °C), interactions between model drugs and the silk protein structure again impact sample degradation. Just as in the fibers, silk fibers without model drugs degrade the fastest with rifampin, alcian blue, and indigo carmine showing the most stabilization based on smaller derivative peaks in Figure 4b.

Another interesting note specific to films is the appearance of extra degradation peaks in rifampin films. Rifampin is a drug that must be kept in cold storage, rendering it sensitive to heat with a low degradation temperature of 183 °C [47]. This degradation is clearly seen as a sharp peak in Figure 4b, but fiber samples with the same amount of rifampin do not have the same peak in Figure 3b. Because of protein-model drug interactions, plus the porous 1D geometry of micro-/nanofiber networks, rifampin is protected from thermal degradation by silk fibers, but not in 2D silk films. In nonporous 2D films, rifampin only sits on the film surface without integrating into the protein network, which does not provide the thermal protection that fibrous networks do. Previous studies [6,11,48] also show the ability of fibers to provide thermal protection for therapeutic loads.

**Figure 5:**
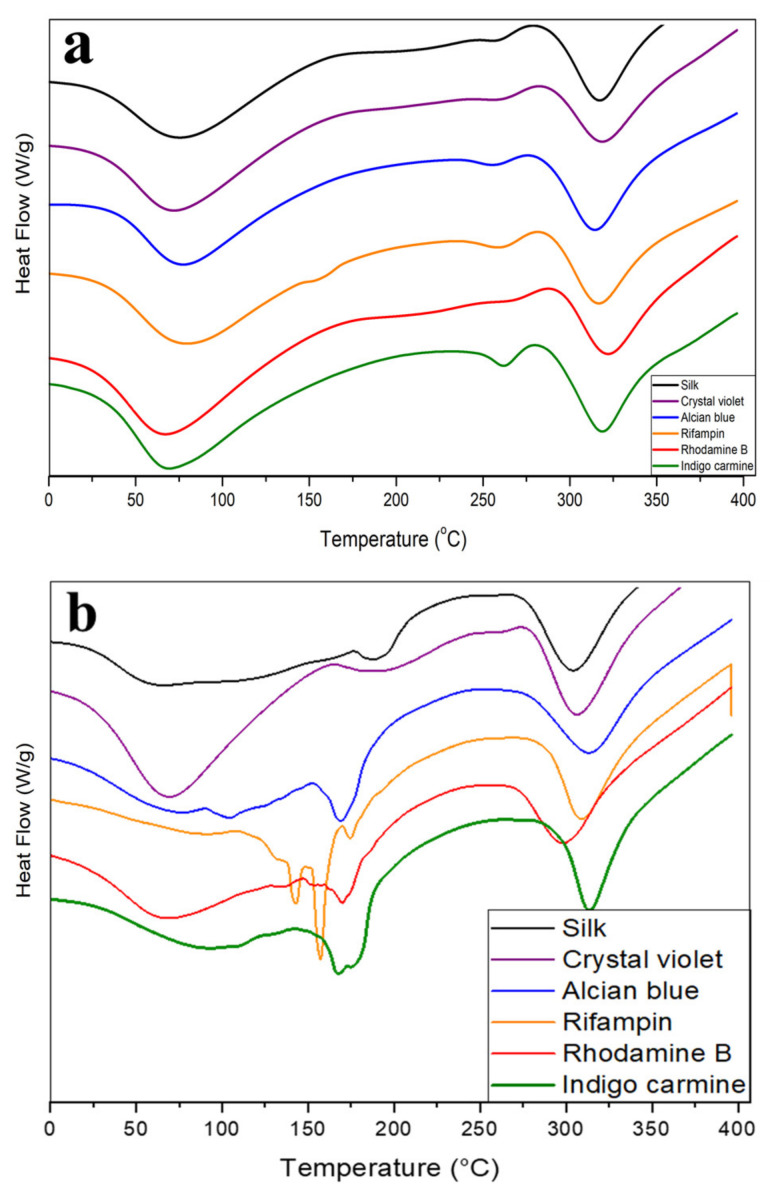
DSC total heat flow thermograms of silk-model drug fibers and films. Total heat flow graphs of silk and silk-model drug (**a**) air-spun fibers and (**b**) thin films. Exothermic is upward.

Further thermal analysis was done using DSC (Figure 5 and Table 1). All fiber samples contain solvents embedded in the micro-/nanofiber network, even after drying, resulting in the first endothermic peaks centered around 50 °C as the excess solvent evaporates (Figure 5a). This correlates well with TGA thermograms where silk fibers saw an initial 5–10% mass loss as the bound solvent evaporated. The magnitude of these peaks is roughly the same in all samples, but slightly higher in rifampin, rhodamine B, and indigo violet silk composite fibers, which were all shown to interact with silk protein structure in the FTIR analysis. These interactions may have shifted the protein structure enough to allow more solvent into the fiber network to bind to the protein. Between 250–350 °C all samples see endothermic peaks as the fibers begin to degrade. This can be cross referenced against TGA thermograms, where major degradation largely occurs in the 300–350 °C region. Rifampin fibers see an addition peak around 155 °C that the other samples do not, likely related to the degradation of rifampin.

Total heat flow graphs of silk-model drug films are much noisier than their fiber counterparts, again supporting the hypothesis that model drugs are not able to interact well with the protein structure of silk within the 2D film geometry. Rifampin, alcian blue, indigo carmine, and rhodamine B all saw steep degradation at 150 °C during the TGA analysis losing as much as 0.5%/°C; this correlates to the noisy heat flow thermograms centered around 150 °C in Figure 5b for these samples. For some drugs with lower melting points, such as alcian blue (T_m_ = 148 °C) and rifampin (T_m_ = 183~188 °C), their degradation peaks may also be mixed with the melting behavior of the drugs. Because the model drugs are unable to interact with 2D geometry, silk thin films are unable to provide thermal protection for the model drugs in the way fibers did. Crystal violet is the only exception to this, with a thermogram profile similar to its fiber counterpart, possibly due to its small size. Crystal violet also contains a protonated amine, which could interact with the carboxylic acids in the amino acids of silk. Despite lacking the porous 1D geometry of micro-/nanofibers, crystal violet may be a small enough molecule to infiltrate the 2D fiber network and interact with the protein structure.

**Figure 6:**
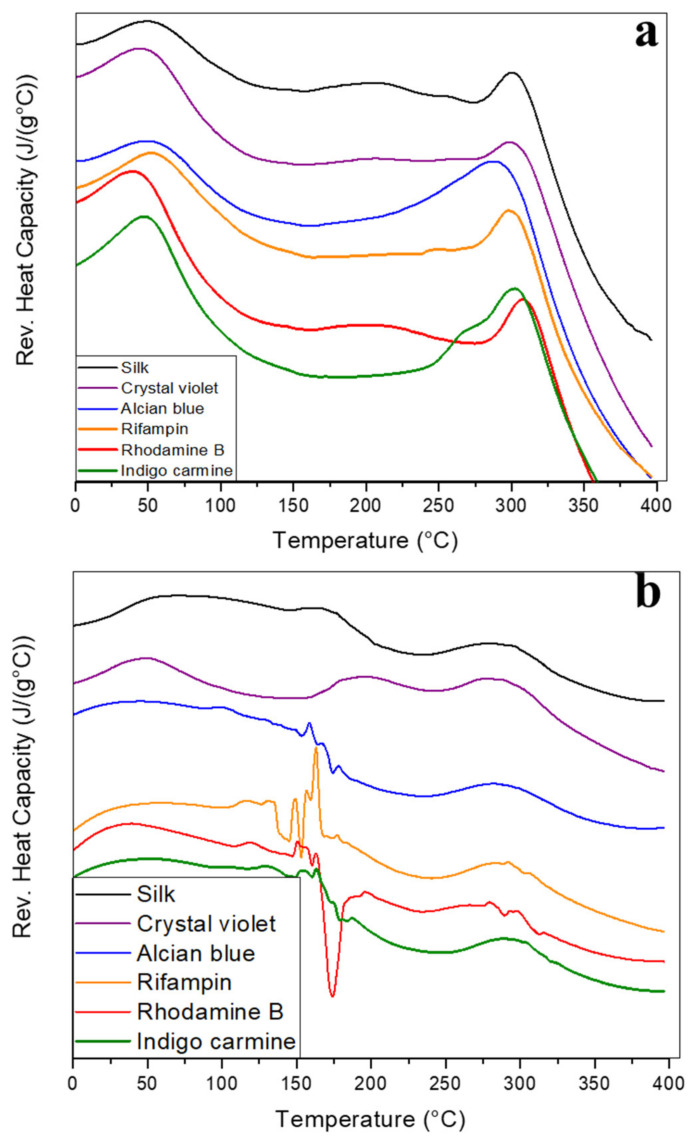
DSC reversing heat capacity thermograms of silk-model drug fibers and films. Reversing heat capacity of silk and silk-model drug (**a**) air-spun fibers and (**b**) thin films. Exothermic is upward.

Glass transition (T_g_) temperatures can be identified via reversing the heat capacity thermograms on fiber samples (Figure 6a), although they are obscured by some of the model drugs. Pure silk has the most obvious T_g_ identified by the endothermic peak between 200–225 °C. Rhodamine B and crystal violet have less discernable T_g_ peaks, followed by alcian blue. In rifampin and indigo carmine embedded fibers, the T_g_ cannot be identified. Since rifampin degrades/melts at 183 °C it is likely that drug degradation obscures reversing heat capacity changes caused by a glass transition. Indigo carmine and rifampin fibers retained a higher mass before major degradation and rhodamine B showed a slower, steadier decomposition during the TGA analysis. This shows that these model drugs can impact the thermal integrity of silk fibers, and likely influences the visibility of T_g_ in reversing the heat capacity thermograms.

The reversed heat capacity graphs of silk-model drug films (Figure 6b) are, as with the heat flow graphs, noisy. Regardless, some numerical information can still be extracted from them. In all samples, there may be an early T_g_ at 50 °C caused by plasticization of the internalized solvent inside the films. In drug-embedded samples, this T_g_ peak is less clear, although again crystal violet has the least obscuring effect. Alcian blue, rifampin, and indigo carmine obscure this transition so much that it is indistinguishable. The thermogram then advances into the noise region where most of the drugs degrade, causing noisy signals and obscuring any T_g_ signals. There is a possible T_g_ in crystal violet and pure silk samples at 175 °C, but all other embedded samples are too noisy to distinguish if a second T_g_ occurs. This once again confirms that the 2D film geometry is unable to protect embedded model drugs from thermal degradation, but the 1D porous geometry of fibers provides protection for the embedded model drugs. Past this noisy region, all samples see another decrease in heat capacity when major degradation begins around 300 °C.

### 2.3. Morphology Discussion

**Figure 7:**
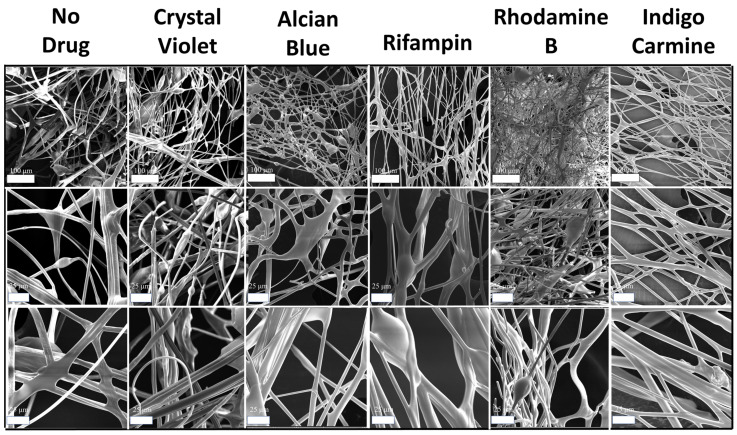
SEM images of silk-model drug air-spun fibers. First row scale bars are 100 μm. Second and third row scale bars are 25 μm.

The morphology of silk-model drug micro-/nanofibers and thin films was studied using SEM. Figure 7 depicts SEM images of silk fibers both with and without embedded model dugs at various magnifications (100, 500, 1000×). Qualitative analysis shows that all samples maintain their porous network. Although some aggregates are visible, the majority of the fibrous network is unaffected by the incorporation of the model drugs. This indicates that at low weight percentages of drugs, micro-/nanofibers maintain their beneficial morphology, making them valuable candidates for drug delivery vehicles. Quantitative analysis was also completed using ImageJ software. Measurements of the fiber diameters revealed an average diameter of 1–9 μm, indicating that the air-spraying method was capable of producing small, submicrometer scale fibrous networks. Additionally, the incorporation of model drugs did not impact the average diameter of the fibers.

**Figure 8:**
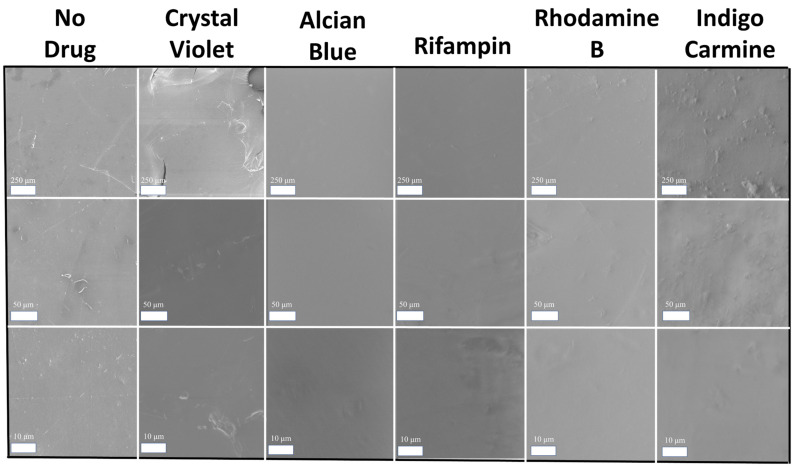
SEM images of silk-model drug thin films. Scale bars from top to bottom row: 250 μm, 50 μm, 10 μm.

SEM analysis was also performed on silk and silk-model drug thin films for comparison. Images of the film surfaces were taken at 100, 500, and 2000× magnification (Figure 8). All samples are smooth with very little, if any, aggregates of model drugs visible. The roughest films are the indigo carmine-embedded films, which also formed rough films in corn zein-based samples in another study [6]. Indigo carmine is the only anionic model drug in this sample set, which might indicate that different electrostatic interactions occurred during fabrication to influence the surface morphology of the films. Regardless, all film samples are still sufficiently smooth to be handled as topical drug delivery patches.

### 2.4. Drug Release Testing

**Figure 9:**
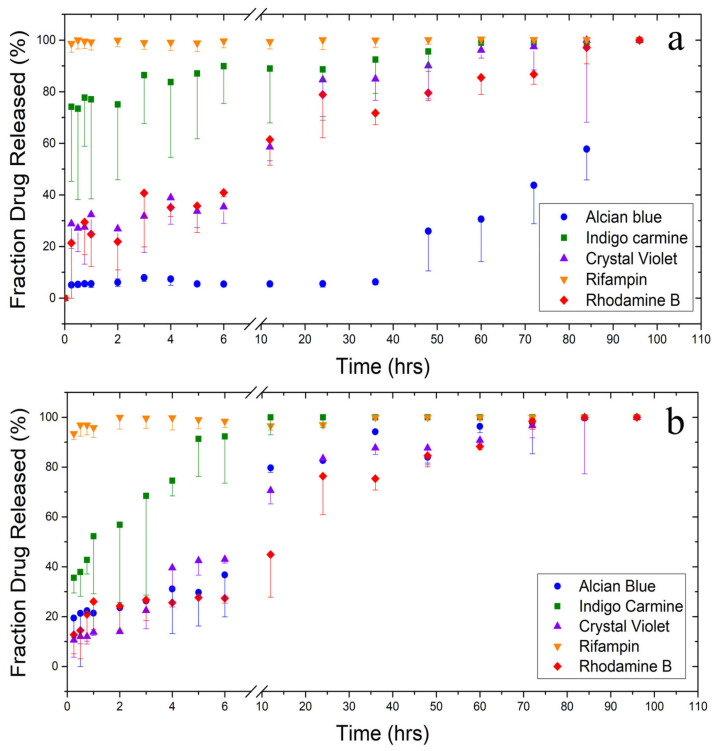
Release profiles from silk-model drug fibers and films. Normalized release profiles of various model drugs from silk (**a**) air-spun fibers and (**b**) thin films. Error bars represent standard deviation.

After seeing the varied effects of biomaterial geometry on protein-drug interactions in FTIR and thermal analysis, a drug release test was performed to see if the choice of fiber or film resulted in a significant difference in release profiles, as well as to see the release profile of various drugs and model drugs from the silk biomaterials (Figure 9 and Table 2). The model drugs chosen for this test were reflective of many pharmaceuticals in the US pharmacopeia while providing a range of molecular weights, hydrophobicity, solubilities, and charges (Table 3). Some compounds are also in use medically already; rifampin has historically been used to treat tuberculosis [49] and has more recently been used to treat atopic dermatitis [50]. Rifampin and crystal violet also show anti-fungal and anti-bacterial activity in several studies [51,52]. Because of frequent off-target effects [53,54,55], the oral use of rifampin is discouraged, leading to the need for localized delivery methods, including topical delivery. All model drugs used here also absorb light in the UV-visible spectrum, allowing for easy measurements of their release from biomaterials. A two-sample T-test was also performed between fiber and film groups for each model drug in order to test for significant differences in release profiles as a result of biomaterial geometry with an alpha significance level of 0.05.

Figure 9 depicts the normalized release of model drugs from (a) fibers and (b) films based on the amount of drug released at 96 h. Beyond this time point no significant release of drugs was observed. One property of the model drugs that helps us understand their biochemical interactions and their release kinetics is hydrophobicity. Hydrophobicity can be quantified using log P, which is a ratio of a molecule’s solubility in octanol (organic phase) to its solubility in water (aqueous phase). This is not to be confused with the *p* value reported here, which is a statistical probability of a difference in sample means. Alcian blue shows the slowest release out of all the model drugs. With a log P of −9.7, alcian blue is the most hydrophilic molecule, but it also has the lowest water solubility and is by far the heaviest molecule. Biomaterial geometry also has the most obvious effect on drug release for this molecule with a *p* value of 0.00. In alcian blue-embedded silk fibers, there is a very slow, controlled release of the drug. Fibers are able to retain 93% of their alcian blue load over 36 h, compared with films which have already released 19% of their alcian blue load in the first 15 min. Once the release of the drug passes 50%, however, release speeds up. This is likely due to drug–drug interactions [11], which help the drug resist detachment from the silk protein structure. When coupled with its low solubility in water (1 mg/mL at 25 °C [6]) and the hydrophobic effect which helps silk preferentially fold its hydrophobic amino acids inside itself, the result is a slow release of alcian blue from fibers until enough of it has been released that it can no longer interact with itself or the silk protein structure. More evidence of this interaction can be seen in the post-soak FTIR spectrum of alcian blue fibers (Figure 1b). Alcian blue prevented silk from shifting towards intermolecular beta sheet structure more than any other drug, implying that its size and hydrophobicity may have affected protein folding while protecting itself from water. Since film samples did not see this same interaction in the FTIR (Figure 2b), it did not interact with the silk protein structure to slow its release into solution.

Rifampin, which is the most hydrophobic molecule with a log P of 2.77, saw an almost immediate release from both fiber and film samples. Regardless, silk films do have a significantly slower release than silk fibers do (*p* = 0.013). This is likely due to the large surface-area-to-volume ratio of micro-/nanofibers, which exposes the surface of the fibers to water more than the surface of films to enhance mass transfer between the fibers and the water [4]. The model drug with the next second log P (log P = 1.01), and the second quickest release is indigo carmine. Out of all the hydrophobic model drugs (log P > 0), indigo carmine is the least hydrophobic. For this molecule, there is not a significant difference in release profiles between fibers and films (*p* = 0.072). The release of indigo carmine is more prolonged than rifampin, with films retaining the drug for 12 h and fibers for 60 h.

The remainder of the model drugs all have a more sustained release profile. Crystal violet and rhodamine B, which have similar log P values of 1.4 and 1.95, show similar release profiles. Additionally, their release from both fibers and films are comparable (*p* = 0.101 and 0.085 for crystal violet and rhodamine B, respectively). All crystal violet and rhodamine B samples are able to hold on to at least half of their drug load for at least 12 h while soaking in water. After this, the release slows, but there is still a continuous and fairly linear release profile until all of the model drug molecules are released from their fiber or film carrier.

Finally, alcian blue shows the slowest release out of all the model drugs. In addition to fitting the hydrophobicity trend with a log P of −9.7, alcian blue also has the lowest water solubility and is by far the heaviest molecule. Biomaterial geometry also has the most obvious effect on drug release for this molecule with a *p* value of 0.00. In alcian blue-embedded silk fibers, there is a very slow, controlled release of the drug. Fibers are able to retain 93% of their alcian blue load over 36 h, compared with films which have already released 19% of their alcian blue load in the first 15 min. Once the release of the drug passes 50%, however, release speeds up. This is likely due to drug–drug interactions [11], which help the drug resist detachment from the silk protein structure. When coupled with its low solubility in water (1 mg/mL at 25 °C [6]) and the hydrophobic effect which helps silk preferentially fold its hydrophobic amino acids inside itself, the result is a slow release of alcian blue from fibers until enough of it has been released that it can no longer interact with itself or the silk protein structure. More evidence of this interaction can be seen in the post-soak FTIR spectrum of alcian blue fibers (Figure 1b). Alcian blue prevented silk from shifting towards intermolecular beta sheet structure more than any other drug, implying that its size and hydrophobicity may have affected protein folding while protecting itself from water. Since film samples did not see this same interaction in the FTIR, it did not interact with the silk protein structure to slow its release into solution.

The Pearson correlation coefficient is also reported for all samples in order to see how strong the linear relationship is between fiber samples and film samples. Indigo carmine-, crystal violet-, and rhodamine B-embedded samples all showed a very strong linear relationship with Pearson correlation coefficients of 0.914, 0.971, and 0.979, respectively. This indicates, over the course of the release testing, that the amounts of drug released by either fiber or film samples are comparable with one another. This is a sensible result, since none of these model drugs showed a significant difference in release kinetics based on *p* values. Alcian blue and crystal violet, however, showed much lower linear correlations of 0.645 and 0.488, respectively. This indicates a nonlinear relationship in the amount of drug released between each sample set. Since alcian blue and crystal violet had statistically different release kinetics based on *p* values, it makes sense for there to be poor linear correlation between the fiber and film sample sets for each drug. All sample sets showed a positive correlation, which is sensible since the cumulative amount of drug released increases over time for all samples.

### 2.5. Concept of Interaction

**Figure 10:**
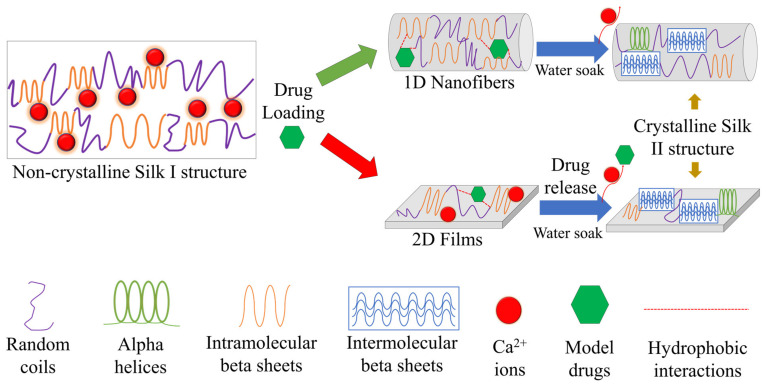
Conception of interaction. Porous 1D fiber networks allow for the integration of model drugs, which then influence the self-folding behavior of silk protein. In flat 2D films, model drugs are unable to interact in the same way. As a result, fibers are able to protect embedded model drugs from thermal degradation and slow the release of the molecules from its structure. In thin films, the drugs are more susceptible to thermal degradation and there is less control over their release.

Silk provides a promising platform for the delivery of various therapeutic molecules based on this study using model drugs. In particular, the porous 1D micro-/nanofiber geometry or flat 2D thin film geometry, coupled with the physicochemical properties of the therapeutic load, create a platform for fine-tuned drug release. The porous 1D micro-/nanofiber geometry also allows for several molecules to infiltrate the protein structure of silk, offering protection from thermal degradation (rifampin, rhodamine B, indigo carmine) or slowing its release from the fiber mat (alcian blue). These interactions are similar to native protein folding via the hydrophobic effect. For drug release in an aqueous environment, this becomes especially relatable as silk starts to self-assemble into intermolecular beta sheets while under the influence of the chemistry of the model drugs embedded within the silk fiber network. On the contrary, the flat, 2D geometries of thin films do not offer the same benefits as a drug delivery vehicle. Model drugs are not able to infiltrate the protein structure or have a strong influence on the self-folding of silk. This results in less thermal stability and lower control over the release profile of embedded therapeutics. Figure 10 is drawn to illustrate these biophysical and biochemical interactions. The results of this study indicate that diffusion mechanisms alone do not dictate drug release; when designing a drug delivery vehicle, especially a protein-based biomaterial, biophysical and biochemical interactions are also important. These results are comparable to other studies utilizing silk as a drug delivery vehicle [34,35] where silk II crystals were found to be important regulators in the release of model drugs.

## 3. Materials and Methods

### 3.1. Materials Preparation

Bombyx *mori* silk cocoons were purchased from Treenway Silks. To remove sericin, cocoons were boiled in a 0.02 M NaHCO_3_ (Sigma-Aldrich, St. Louis, MO, USA) solution for 15 min, followed by four washes in deionized water for 15 min each yielding silk fibroin fibers. Before further use, the fibers were dried in an oven at 60 °C overnight. Formic acid of ACS 98% grade was purchased from EMD Millipore corporation (Burlington, MA, USA). ASC grade calcium chloride (CaCl_2_) from AMRESCO (Solon, OH, USA) was used to create a 4% wt./vol formic acid CaCl_2_ solution to dissolve silk. The following model drugs purchased from VWR International (Radnor, PA, USA) were used as-is: crystal violet, indigo carmine, alcian blue 8GX, rhodamine B, and rifampin.

### 3.2. Silk Fibers

**Figure 11:**
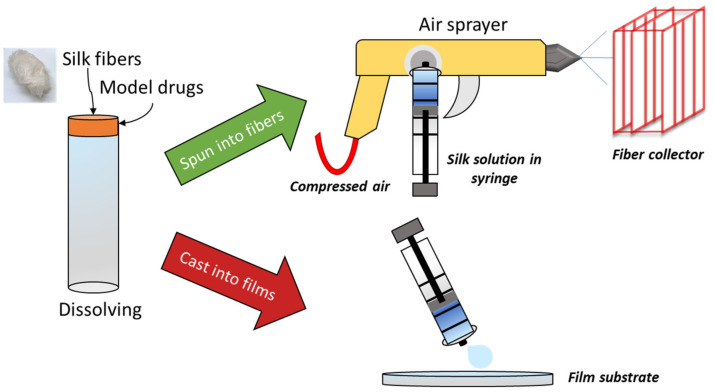
Synthesis of silk biomaterials for drug delivery. Silk-model drug solutions were spun into micro-/nanofibers using a spray gun or cast onto PDMS molds for film fabrication.

Silk submicrometer fibers were fabricated by dissolving 0.75 g of silk fibers into 5 mL of 4% CaCl_2_ formic acid solution. Fibers were kept in the oven at 60 °C until ready to dissolve, removing moisture and so helping them to dissolve. This concentration was chosen based on strong mechanical and thermal strength obtained in prior studies [31]. While higher concentrations of silk solutions are possible, they result in viscous solutions that clog the spray gun. In samples that contained model drugs, 0.05 g of the appropriate drug was dissolved into the formic acid solution prior to dissolving silk. A benchtop vortexer helped dissolve the silk initially. Centrifugation at 2000 RCF for 10 min helped dissolve the silk and separate any undissolved clumps from the solution prior to spraying. Once dissolved and absent of clumps, the silk or silk-model drug solutions were transferred to a syringe and sprayed through a high-pressure low volume (HPLV) gravity-feed spray gun onto a grated substrate from a distance of about 20 cm away. The spray gun was supplied with ultra-dry air at 80 psi. Knobs on the spray gun were used to fine-tune the spraying process for the best results; typically, the smallest spray angle was used with a lower fluid intake and pressure. Ambient humidity varied between 20–22% during air spraying. After spraying, the fibers were collected and dried in a vacuum oven at 60 °C for 12 h to remove excess solvent that did not evaporate off during fabrication. A summary of the fabrication process is shown in Figure 11.

The variety of model drugs was chosen to represent the drugs in the pharmacopeia. There is variation among chemical and physical properties including hydrophobicity, molecular weight, solubility, and melting point as summarized in Table 3. Model drugs also provided the benefit of having distinct, measurable absorbance at distinct wavelengths of UV-vis light for easy measurement of release kinetics from the biomaterials. Only a small amount of each drug was needed to provide a measurable absorbance, which meant a lower concentration could be used to prevent model drug crystals from clogging the spray gun.

### 3.3. Silk Films

2D silk films were also fabricated to compare the effect of biomaterial morphology on the release kinetics of the model drugs (Figure 11). Solutions were prepared in the same way as those prepared for fibers. A 3 mL of silk or silk-model drug solution was poured onto circular PDMS molds and dried at room temperature for 48 h to allow films to form. Once formed, films were dried in a vacuum oven at 60 °C for 12 h to evaporate excess formic acid prior to any characterization.

### 3.4. Morphology Characterization

The morphology of silk fibers and films was characterized using scanning electron microscopy (SEM) images obtained with a Leo 1530 VP SEM. Images were taken at 100, 500, 1000, 2000, and 5000× magnification at an EHT of 5.00 kV. Prior to imaging, samples were sputter coated in gold to improve their conductivity. ImageJ image processing software was used to measure the average diameter of fiber samples.

### 3.5. Fourier Transform Infrared Spectrometry (FTIR)

Further morphology analysis was completed using Fourier-transform infrared spectrometry (FTIR). The spectra were obtained using a Bruker Tensor 27 Fourier-transform infrared spectrometer fitted with a deuterated triglycine sulfate detector and multiple-reflection horizontal MIRacle attenuated total reflection (ATR) attachment (with Ge crystal). All readings were taken between 4000 and 400 cm^−1^ at a resolution of 4 cm^−1^, 64 sample scans, and 64 background scans. At least two readings were taken on each side of each sample to ensure homogeneity, but only one spectrum is shown from each sample. The ATR crystal was cleaned with methanol between samples.

### 3.6. Differential Scanning Calorimetry (DSC)

Differential scanning calorimetry (DSC) was performed using a Q100 DSC (TA Instruments) purged with nitrogen gas at a rate of 50 mL/min and equipped with a refrigerated cooling system. Prior to use, calibration was performed with indium for heat flow and temperature; heat capacity was calibrated using aluminum and sapphire reference standards. About 5–7 mg samples of fibers and films were encapsulated in aluminum pans and temperature-modulated DSC (TMDSC) was performed at a heating rate of 2 °C/min, with a modulation period of 60 s and amplitude of 0.318 °C. Readings were taken from −40 to 400 °C.

### 3.7. Thermal Gravimetric Analysis (TGA)

Further thermal analysis was performed using an SDT-Q600 TGA (TA Instruments) with nitrogen purge gas flowing at a rate of 100 mL/min. The mass change of small samples (4–6 mg) of fibers and films was measured as they were heated from 25–800 °C at a rate of 10 °C/min.

### 3.8. Drug Release Study

The release of model drugs from samples was stimulated by immersing them in 40 mL of deionized water. Small (~6 mg) samples of silk fibers and films with and without model drugs were prepared in triplicate. At set time points over four days (15 min, 30 min, 45 min, 1 h, 2 h, 3 h, 4 h, 5 h, 6 h, 12 h, 24 h, 36 h, 48 h, 60 h, 72 h, 84 h, and 96 h) 200 µL of solution was removed from each sample solution and placed into a 96 well plate for later analysis. After sampling, each solution was restored to its initial volume by adding 200 µL of deionized water back to the sample solutions. Well plates were covered between sampling to prevent loss or contamination of solutions. Once all aliquots were collected, the UV absorbance was measured using a SpectraMax i3x plate reader (Molecular Devices LLC). Ten readings were taken horizontally across each well with spacing of 0.57 mm between each reading. After outliers were removed, the remaining values were averaged for each time point and normalized from 0 to 1 for comparison between model drugs and between fibers and films. After release testing, all samples were dried in the oven at 60 °C for 12 h to remove water before being re-analyzed by FTIR.

### 3.9. Statistical Analysis

To better quantify any differing release kinetics between fibers and films, a paired two-sample *t*-test was performed using Excel software. The null hypothesis for the analysis was that the mean amount of drug released from the fibers was the same as the mean amount of drug released from the films. A *t*-test was performed for each model drug used. From the 17 time points measured, there were 16 degrees of freedom for the *t*-test. Any statistical difference was determined by comparing the t-statistic generated for each test to the t-critical value and using the *p* value, the probability of the null hypothesis being true. A two-tailed alpha significance level of 0.05 was chosen for all samples. When *p* < 0.05, the release speeds between fiber and film samples was deemed significantly different. The Pearson correlation coefficient was also calculated during each *t*-test to observe any covariance between each sample set.

## 4. Conclusions

This study utilized a novel fabrication method to create model drug-embedded silk micro-/nanofibers with high throughput efficiency and without the need for a high voltage power source by using air-spraying. Analysis showed that the porous 1D geometry of fibers provided a better network for the model drugs to interact with the protein structure of silk in order to change the release speed of the model drugs in an aqueous solution. The physicochemical properties of the model drugs proved to be important in tuning the release speed; the hydrophobicity of the model drugs was especially important. Silk protein utilizes the hydrophobic effect to self-assemble into intermolecular beta sheets. When model drugs are embedded in the silk protein structure, the hydrophobicity of the model drugs can impact the degree of self-assembly in fibers, but not in films. As a result, model drugs have a more controlled release from fibers and are provided with thermal protection by the silk micro-/nanofibers. The flat, 2D geometry of films do not allow for these interactions, resulting in less control of the model drug release and lower thermal stability. Further study of the interactions between therapeutic molecules and the protein structure of silk may lead to improved protein-based drug delivery vehicles with fine-tuned drug release.

## Figures and Tables

**Table 1:** Key temperatures from thermal analysis of silk-model drug fibers and films. Values obtained from DSC; temperatures recorded in the middle of the curve for changes in heat flow (middle degradation) or heat capacity.

Sample	Fiber		Film	
	Glass Transition T_g_/°C	Major Degradation T_d_/°C	Glass Transition T_g_/°C	Major Degradation T_d_/°C
Pure Silk	175	299	182	286
Alcian Blue	N/A	298	N/A	292
Indigo Carmine	N/A	302	N/A	302
Rifampin	N/A	301	N/A	297
Crystal Violet	179	299	177	291
Rhodamine B	183	306	N/A	283

**Table 2:** Statistical values from model drug release. All probabilities are compared with a two-tailed t critical of 2.120 and an alpha significance level of 0.05.

Silk with Drugs	Pearson Correlation	T-Stat	*p* (T ≤ t)
Alcian Blue	0.645	−5.781	0.00
Indigo Carmine	0.914	1.929	0.072
Rifampin	0.488	2.792	0.013
Crystal Violet	0.971	1.740	0.101
Rhodamine B	0.979	1.839	0.085

**Table 3:** Physicochemical properties of selected model drugs [6].

Model Drug	Molecular Weight (g/mol)	Solubility in Water at 25 ℃ (g/L)	Log P	Melting Point (°C)
Crystal violet 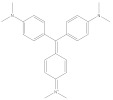	407.98	50	1.4	205
Indigo carmine 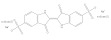	466.36	10	1.01	>300
Rhodamine B 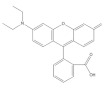	497.02	8	1.95	~210–211
Rifampin 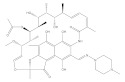	822.94	2.5	2.77	~183–188
Alcian blue (8gx) 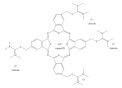	1298.9	1	−9.7	148

## Data Availability

Not applicable.
